# From the Principle of Inertia to the Death Drive: The Influence of the Second Law of Thermodynamics on the Freudian Theory of the Psychical Apparatus

**DOI:** 10.3389/fpsyg.2020.00325

**Published:** 2020-02-28

**Authors:** Jessica Tran The, Jean-Philippe Ansermet, Pierre Magistretti, François Ansermet

**Affiliations:** ^1^Faculty of Biology and Medicine, Université de Lausanne, Lausanne, Switzerland; ^2^Département D’études Psychanalytiques - UFR IHSS, Université de Paris, Paris, France; ^3^Agalma Foundation Geneva, Geneva, Switzerland; ^4^Centre de Recherches Psychanalyse, Médecine et Société, Université Paris Diderot, Paris, France; ^5^Ecole Polytechnique Fédérale de Lausanne, Lausanne, Switzerland; ^6^Institut de L’esprit de Cerveau, École Polytechnique Fédérale de Lausanne, Sion, Switzerland; ^7^Lausanne University Hospital (CHUV), Lausanne, Switzerland; ^8^Faculté de Médecine, Département de Psychiatrie, Université de Genève, Geneva, Switzerland

**Keywords:** Freud, principle of inertia, death drive, thermodynamics, entropy

## Abstract

In the Freudian theory of the psychical apparatus, the introduction from the 1920s onward of the second drive dualism appears as a major turning point. The idea of a “death drive,” first expressed in *Beyond the Pleasure Principle* (Freud, 1920), is generally considered to be a new concept, one that represents a break with Freud’s previous thinking. It has often surprised the scholars because it seemed, at first sight, difficult to reconcile with the idea of the singularity of living organisms within which the psychical functions form an integral part. Our research aims to demonstrate that the theory of the death drive does not represent a complete change in direction for Freud. It is present, in essence, in his earliest work, to the extent that the “principle of inertia” described in 1895 in *A Project for a Scientific Psychology* (Freud, 1895) can be seen as a precursor to the death drive. Based on a reading of Freud’s early formulations of his ideas, we aim to bring to light how certain aporias that seem inherent to the concept of the death drive can be overcome if we consider them in the context of an epistemological model that draws on the paradigms of physics which were conveyed by the Helmholtz School. Namely, we can consider the idea of death drive in reference to the principle of entropy and the laws of thermodynamics.

## Introduction

The establishment of the idea of the “death drive” at the theoretical turning point of the 1920s is often seen as a significant watershed in Freudian theory. A defining moment, that led to profound epistemological reconfigurations, with the advent of a new dualism of the drive. Indeed, in his essay *Beyond the Pleasure Principle* (Freud, 1920) Freud introduces a distinctly subversive theory. If, in his definition of the pleasure principle, he had recognized that “the effort to reduce, to keep constant or to remove internal tension” (Freud, 1920, p. 55) constitutes “The dominating tendency of mental life, and perhaps of the nervous life in general” (Freud, 1920, p. 55), he made this concept even more radical by introducing an apparently paradoxical theory:

“the life process of the individual leads for internal reasons to an abolition of the chemical tension, that is to say, to death.” (Freud, 1920, p. 55)

If this concept can, in some respects, appear to be resolutely surprising, and especially novel in Freudian thinking, a return to Freud’s first theoretical ideas – in particular *A Project for a Scientific Psychology* (Freud, 1895) – shows that this concept existed already in essence as far back as his first theoretical elaborations; most notably in the description of the “principle of inertia.” Indeed, Freud had, as early as 1895, postulated the existence of a first principle within the functioning of the nervous system, a principle that would consist in trying to achieve a “zero level” of excitation: the principle of neuronal inertia is defined as the fact that “neurons tend to divest themselves of (*Q*)” (Freud, 1895, p. 296). The “quantity (*Q*)” here stands for the quantity of neuronal excitation, a theory that constitutes the “first fundamental definition” of *A Project for a Scientific Psychology* (Freud, 1895). Thus, it is not a principle of constancy, or of homeostasis, that would hark back to the general tendency of the organism to maintain a positive optimum level (be it in body temperature, concentration of oxygen in the blood, etc.), that Freud places at the basis of the psychical function. On the contrary, he defines the original tendency of the neuronal system as one of trying to reach a “level = 0”; the equivalent of a search for a total absence of excitation, or of the fastest possible discharge of the *Q* quantities to re-establish a level of zero (Freud, 1895). This hypothesis at the root of the whole Freudian theoretical construction can, in a way, if we follow the arguments of Laplanche and Pontalis “appear to be an aberration from the point of view of the life sciences” (Laplanche and Pontalis, 1967a, p. 326), this in so far as that it postulates a first principle in the functioning of the nervous system which would be “the negation of any stable difference in level” (Laplanche and Pontalis, 1967a, p. 326). Would psychoanalysis therefore be, in its fundamentals, radically estranged from a theory of the living organism? To shed light on this aporia it is necessary to put the definition of “principle of inertia” back into its context, within the radically physicalist epistemological model that Freud draws on; Freud being a faithful heir to the tradition of the Hemholtz school, and its contemporaneous research in thermodynamics (Tran The et al., 2018). More specifically, it is important to consider how certain complexities inherent to the definition of the Freudian principle of inertia, and to the death drive as its logical continuation in the 1920s, can be explained through reference to entropy, that is to say the second law of thermodynamics. Thus, we can observe how a return to the physicalist epistemological foundations of the Freudian theoretic model makes it possible, in a surprising way, to lift the apparent aporias between the presence of a death drive at work in the psyche on the one hand, and on the other the indissociable natures of the link between the psychical function and the living organism within which it occurs.

## The Influence of the Helmholtz School of Physics on Freud’s Training

In order to understand why the death drive might, on first impression, “seem like an aberration from the point of view of the life sciences” (Laplanche and Pontalis, 1967a, p. 326) (translated for this publication), most notably from the point of view of biology and physiology, we need to remember that the dominant epistemological paradigm during the formative years of Freud’s medical training was very different to the “physiological revolution” instigated by Claude Bernard in 1860s France. Bernard’s influence had established physiology as an independent and autonomous discipline in relation to physical chemistry (Tran The et al., 2018). However, it is in a radically different geographic and scientific context that Freud undertook his medical studies, at the Vienna Faculty, in autumn 1873 (Jones, 1953). Beyond the Rhine, physiology’s autonomy with regards to physics was far from complete, and it was within a paradigm that is profoundly antagonistic with regards to that of French biology, that Freud’s training took place. At the end of his third year, Freud joined Ernest Brücke’s physiology laboratory. Freud viewed Brücke as a “model” (Freud, 1925). Besides the respect and admiration that Freud felt for this undisputable master, this filiation bears witness to Freud’s adherence to a whole scientific paradigm to which he would make himself heir. As Jones (1953) underlines, the Brücke Institute had close ties with the Helmholtz School. The story of this scientific movement began in the 1840s, with the friendship between various physiologists trained in Johannes Müller’s theories of specific nerve energies (Assoun, 1981). Du Bois-Reymond, Brücke, Helmholtz, and Ludwig were medical men who appear to have been driven by a veritable “spirit of crusade,” who, as Du Bois-Reymond tells it, had undertaken to “pledged a solemn oath to put into effect this truth: “No other forces than the common physical-chemical ones are active within the organism.” (Jones, 1953, p. 40). Although they were all medical men by training, their scientific ideas were completely subordinated to the science of physics. This little group, augmented by the arrival of new members, young physics and physiology students who opposed vitalism, became in 1845 the Berliner Physikalische Gesellschaft, the Berlin Physics Society (Jones, 1953). In less than 30 years, they came to dominate the German scientific landscape, becoming the most influential professors of medicine and physiology of the time, and in their turn training a whole generation of students; amongst them Freud and Wundt. Thus, it was a practice characterized by its diversity and its lack of specialization that Freud inherited during his years of training at the Brücke Institute. However, it was physics that represented for all those related disciplines the epistemological model *par excellence*. We can see here that the German school of physiology positioned itself within a movement exactly the reverse of Bernardian physiology. Whereas in France there is a demand for a certain independence for physiology, as a science in its own right, autonomous from physics. The Berlin medical practitioners sought, on the contrary, to subordinate physiology to physics, making the former an offshoot of the latter. It is then, a physiology radically subordinated to physics as foremost dominant science – to which all natural phenomena must be reduced, including those relating to living organisms – that Freud would make himself heir. If it is within this framework that Freud received his training at the Brücke Institute, the major influence exerted by Helmholtz needs to be underlined. Of all the scientist at the Berliner Physikalische Gesellschaft, Helmholtz was, without doubt, the most eminent. Freud saw him as one of his idols, and would regret all his life not having met him in person (Jones, 1953).

In particular, Helmholtz upheld an understanding of nature in terms of mechanics, and the majority of the physiologists of the powerful German school (Liebig, Ludwig, Müller, Du Bois-Reymond, Virchow, and Brücke) adopted his view according to which “the physical-chemical functioning of the living being is subject to the same laws as inanimate matter, and must be studied on the same terms” (Prigogine and Stengers, 1979) (translated for this publication). It is therefore, under the influence of this theoretical framework of an essentially physical epistemological model (and not physiological in the Bernardian sense) that the first ideas expressed by Freud regarding the principle of inertia would develop, when he was writing *A Project for a Scientific Psychology*. Freud (1895). The radically physicalist scientific environment of his years in medical training would leave an enduring mark on the whole of Freud’s corpus. We can see this right up to his later work on the death drive at the turn of the 1920s.

## The Principle of Neuronal Inertia in *A Project for a Scientific Psychology*

The manuscript of *A Project for a Scientific Psychology* (Freud, 1895), written by Freud in September 1895, can be seen as establishing a continuation with the “Theoretical Considerations” that Breuer had contributed to *Studies on Hysteria* (Freud and Breuer, 1895). This work, that remained unpublished during Freud’s lifetime, explicitly expresses the intention to “furnish a psychology that shall be a natural science” (Freud, 1895, p. 295) by describing the psychical processes in terms of “quantitatively determinate states of specifiable material particles” (Freud, 1895, p. 295), neurons – in order to make these processes “perspicuous and free from contradiction” (Freud, 1895, p. 295). Thus, Freud (who like his colleague Breuer views the psychopathological phenomenon of hysteria as an excessive excitation that is impossible to discharge via the usual outlets) will also attempt an explanation in terms of neurophysiology. He does this through a description of the structure and functioning of the nervous system, or “neuronal” system. It was therefore, initially, for the use of neurologists that this project for a scientific psychology was intended. Consequently, Freud retains in his text the energetic and quantitative reference to nervous excitation, which he calls “neuronal excitation”; but abandons the distinction established by Breuer between a “quiescent” energy (the intracerebral tonic excitation), and a “kinetic” energy. This manuscript text, that is clearly neuropsychological in outlook, reveals the first Freudian principles of the regulation of the nervous system. However, when Feud abandons the biological, anatomical, and structural point of view of *A Project for a Scientific Psychology* (Freud, 1895) in favor of the topographical view point of his metapsychology [when he begins Chapter 7 of *The Interpretation of Dreams* (Freud, 1900)], he retains to a great extent his reference to the principles of regulation of the psychical function that he had defined – although their designations will evolve throughout his work.

Committed to the epistemological model of the Helmholtz School, Freud attempts in his project for a scientific psychology to apply the principles of physics to what he terms the “quantity (*Q*)” of neuronal excitation [fundamental idea of *A Project for a Scientific Psychology* (Freud, 1895)]. This quantity (*Q*) that according to him is a “quantity in a state of flow”: “regarded as (*Q*), subject to the general laws of motion” (Freud, 1895, p. 295). It is therefore, before any reference to contemporary thermodynamics, primarily to Newton’s general theory of motion, that is to classical dynamics, that Freud is referring when he introduces his approach. There exists an obvious intertextuality between the first two parts of the general layout of *A Project for a Scientific Psychology* (Freud, 1895), and the beginning of Newton’s *Principia mathematica* (Ansermet, 2019).

In his *The Mathematical Principles of Natural Philosophy* (Newton, 1687/1846), Newton lays down the basis of mechanics, by defining the three laws of motion – a founding act at a turning point in the development of modern science (Ansermet, 2009). Prior to stating his three laws, Newton introduces two fundamental definitions: the first, the “quantity of matter” (Newton, 1687/1846, p. 73), and the second concerning the “quantity of motion” (Newton, 1687/1846, p. 73), are described on the first page of his treatise. In *A Project for a Scientific Psychology* (Freud, 1895), Freud posits as the “first fundamental idea” the concept of “quantity,” which corresponds to the neuronal excitation, and defines it from the outset as a “quantity in a state of flow” (Freud, 1895, p. 295). He is, therefore, choosing to “furnish a psychology that shall be a natural science” (Freud, 1895, p. 295) by representing the psychical processes as a “quantitatively determinate states of specifiable material particles” (Freud, 1895, p. 295): neurons. These neurons, which are isolated one from the other, are traversed by quantities of excitation that submit “to the general laws of motion” (Freud, 1895, p. 295). We will recall that as far back as his communication on histology given in 1882, Freud had already defined the neurons as being “isolated routes of conduction” (Freud, 2017).

Having once defined this first fundamental idea of “the quantity of neuronal excitation in motion,” Freud goes on to describe a primary and absolutely fundamental principle of the neuronal apparatus. A principle that would regulate the movement of the quantities of excitation (that is to say, their circulation, or their flow, in the neuronal apparatus): the “principle of inertia of neurons.” The term “inertia” is totally new to Freud’s writings, in so far as that it does not appear either in *Studies on Hysteria* (Freud and Breuer, 1895), or in his correspondence with Fliess. Henceforth, the principle of neuronal inertia is defined in these terms, “neurons tend to divest themselves of *Q*” (Freud, 1895, p. 296).

When Freud introduces the second fundamental idea of *A Project for a Scientific Psychology* (Freud, 1895), the “theory of neurons,” he will seek to combine what he terms a “theory of quantity” – such that the quantity of neuronal excitation is in motion, and thus regulated by the “general laws of motion” – with his knowledge of neurons as he had observed them in the course of his research in histology at the Brücke Institute. Thus, to these considerations coming from physics are added some anatomical views, seen by some as hypothetical, on the structure and the functioning of the nervous system: “we arrive at the idea of a ‘cathected’ neuron (*N*) filled with a certain quantity (*Qi*), though at other times it may be empty.” (Freud, 1895, p. 298). Neurons could therefore be traversed by some form of “current,” and be, in accordance with the view already expressed in 1882, “routes for conduction.” From there Freud postulates the existence of two types of neuron, sensory neurons and motor neurons, that would enable the nervous system to counteract the reception of quantities by getting rid of them through a reflex motion that discharges the quantity of excitation. It is important to underlined that, contrary to Breuer, Freud does not therefore propose a principle of constancy as the primary tendency of the nervous system. Similarly, he does not refer back to a general tendency of the organism to maintain a positive optimum level (be it body temperature, the concentration of oxygen in the blood, etc.), rather he defines the primal tendency of the neuronal system as the search for a “level = 0” (Freud, 1895). Whereas the Breuerian constancy was a search for an optimum physiological functioning of the nervous system that involved an available, but not excessive, quantity of positive tonic energy, Freud proposes a principle based on physics. The “inertia” at the root of his system is grounded in a search for a total absence of excitation, or the fastest possible discharge of any quantities of excitation so as to restore a level of zero.

If “constancy” might appear to be a physiological term, Freud’s deliberate choice to give the term from physics of “inertia” to this fundamental principle of the psychical function, is not without significance. When associated with the reference to the “general laws of motion,” it is explicitly positioned within the epistemological framework of Newtonian dynamics (Freud, 1895). In his *Principia Mathematica*, after having defined the quantity of matter and the quantity of motion, Newton states the three axioms that make up the “laws of motion.” The first law, termed law of inertia (Ansermet, 2009), is defined in these terms:

“Every body perseveres in its state of rest, or of uniform motion in a right line, unless it is compelled to change that state by forces impressed thereon.” (Newton, 1687/1846, p. 83)

It is relevant to underline that, from a formal point of view, the Freudian argument mimics the structure of Newton’s text: definition of the fundamental theory of quantity (the neuronal excitation in motion), followed by a description of the laws or principles that regulate the movement of the quantities – the principle of inertia appearing as the first axiom. Furthermore, the choice of the term “inertia” posits, from an epistemological point of view, the perspective adopted by Freud as explicitly physicalist, through a reference to classical Newtonian dynamics, and that before the slightest references to any strictly organic principle. Finally, if we refer back to the axioms of *Principia Mathematica*, the meaning of the term “inertia” is to be understood in relation to the first law of motion, and more specifically to the first example given: a body at rest stays at rest unless it is acted upon by a force. Thus, the nervous system, if we imagine a mythical primal state that would be the absence of any excitation, should tend to retain that “zero level.”

Newton goes on to give a second law of motion, one that specifies the event wherein a force acts upon a body, therein moving it out of its state of inertia. This second law thus allows the definition of the principles that govern the change in quantity of motion of the system, the body no longer finds itself in the ideal situation of inertia, and is subjected to the action of a driving force: “The alteration of motion is ever proportional to the motive force impressed” (Newton, 1687/1846, p. 83). Now, Freud finds he also has to consider a second principle, a “secondary function,” that describes the functioning of the neuronal system went it can no longer conserve the zero level. If we apply the first two Newtonian laws of motion to the nervous system we could, as did Freud, imagine that it is initially in a state of repose (the “level = zero”); and that it would tend to remain in that state of repose, until the moment when a given force introduces into it a quantity of movement. However, we can remark, as do Laplanche and Pontalis, that “the relationship between Freud’s use of the principle of inertia, and its application in physics, remains quite flexible” (Laplanche and Pontalis, 1967b, p. 340). It remains quite flexible in so far as that in physics inertia essentially consists in a property of bodies in motion, whereas “for Freud, it is not a property of the envisaged motivation, that is to say excitation, but an active tendency of the system within which the quantities move” (Laplanche and Pontalis, 1967b, p. 340). Nevertheless, the principle of inertia does consist in the tendency of the “particles of matter” that are neurons to divest themselves of the quantities of excitation that traverse them, and therefore to return to their state of repose. Finally, the third Newtonian law of motion, according to which “To every action there is always opposed and equal reaction: or the mutual actions of two bodies upon each other are always equal, and directed to contrary parts.” (Newton, 1687/1846, p. 83), could offer some similarities with the Freudian definition of the discharge during the reflex movement. The neurons “neutralizing the reception of *Qi* by giving it off.” (Freud, 1895, p. 296), through the reflex movement that amounts to a mode of discharge:

“A primary nervous system makes use of this *Qi* which it has thus acquired, by giving it off through a connecting path to the muscular mechanism, and in that way keeps itself free from stimulus. This discharge represents the primary function of the nervous system.” (Freud, 1895, p. 296)

The application of the third law could thus be understood in these terms: faced with the introduction of a quantity of excitation considered as a driving force, the conservation of the initial sate of repose or of non-excitation (the application of the principle of inertia), can be assimilated to a reaction that, in Newton’s terms, would be “always equal and opposite” to the action. The introduction of a quantity of excitation into the system, and its discharge through the reflex, are therefore the result of the action of equal quantities acting “in opposite directions.”

If, in the words of Laplanche and Pontalis, the Freudian construct can in some sense “appear to be an aberration from the viewpoint of the life sciences” (Laplanche and Pontalis, 1967a, p. 326), in that it postulates a first principle of the functioning of the nervous system that is “the negation of all stable difference of level” (Laplanche and Pontalis, 1967a, p. 326), it is relevant, in order to dissipate this aporia, to put it back into context within the radically physicalist epistemological model that Freud uses. Freud remains loyal to the tradition of the Hemholtz School, and to contemporary research in thermodynamics. The principle of constancy as it is described by Breuer is explicitly to be situated within the framework of the first law of thermodynamics, that of the conservation of energy. Thus, according to him, the nervous system would endeavor to keep constant an optimum level of tonic energy to ensure its smooth functioning. However, the Freudian principle of inertia, and the death drive that became its logical continuation in the 1920, could be explained by reference to entropy, that is, to the second law of thermodynamics.

## From the First Law of Thermodynamics on the Conservation of Energy, to the Formulation of the Second Law

The principle of the conservationof energy, based on the work done on heat machines by Carnot [described in his 1824 memoir *Reflections on the Motive Power of Heat.* (Carnot, 1824/1897)], made it possible to formulate an equation for the transformation of heat into a quantity of motion. However, this research was based on the model of an idealized machine, whose utopian output would not be subject to any loss. The beginnings of thermodynamics had therefore neglected to take into consideration the fact that what steam engines consume, irreversibly disappears; no heat machine will restitute the coal it devours. The formulating of the second law of thermodynamics thus stems, according to Prigogine and Stengers (1979), from the transition between a formalization of the transformation of energy within a reversible equation, to the reality of the losses that this conversion entails. According to the law of the conservation of energy, the mechanical work produced and the reduction in the difference in temperature are thus connected in an ideal way through a reversible equivalence, in so far as that the same machine, working in reverse, could restore the initial difference. The taking into account of the losses that, for any real engine, result in an output inferior to the ideal output predicted by this equation, signals therefore the advent of a new science. A science that is no longer based on idealization, but on nature itself, including its “losses.” In this way, the concept of “irreversibility” makes its appearance in physics: there are *irreversible* disturbances, losses that diminish the output of heat machines, which are linked to a dissipation of energy (Laplanche and Pontalis, 1967a).

In 1852 William Thomson formalized this observation by stating the second law of thermodynamics in his papers on the *Dissipation of Mechanical Energy* (Thomson, 1852, In: Locqueneux, 2009). This law states that, in the course of the production of mechanical work from a heat source, “equal quantities of heat are put out of existence” (Thomson, 1852, In: Locqueneux, 2009). This irreversible dispersal of heat is, in the context of thermodynamic machines, synonymous with a loss of output; something that Thomson presents as a “tendency toward a universal degradation of mechanical energy” (Prigogine and Stengers, 1979, p. 185). According to Prigogine and Stengers, by pronouncing the second law of thermodynamics, Thomson accomplishes “the vertiginous leap from motor technology to cosmology” (Prigogine and Stengers, 1979, p. 186), in so far as that he accomplishes an epistemological revolution that renders the world of Laplace, with its simple conservative and eternal ideal machine, definitively obsolete. Henceforth, the world can be described as a machine within which the conservation of heat in motion could only be achieved at the cost of an irreversible wastage, owing to the dissipation of a given quantity of heat (Prigogine and Stengers, 1979). From this principle follows a new description of the world: “the differences that produce an effect are continuously diminishing within nature” (Prigogine and Stengers, 1979, p. 185), and the world in the course of these conversions of energy “depletes these differences” to finally reach a state of thermal equilibrium where no difference that produces an effect would subsist.

We find here a certain resemblance between the Freudian principle of inertia assimilated to a “negation of all stable level of difference” (Laplanche and Pontalis, 1967a), and the second law of thermodynamics according to which the world tends toward an annihilation of the differences that produce effects, in a search for a state of equilibrium. Furthermore, it should be pointed out that, the second law contributed to giving a new importance to the question of time in physics – whereas the Laplacian world, conceived within its reversible unity, had to some extent, not so much resolved, but pushed aside this question. With the advent of the concept of irreversibility in physics, time also introduces itself into that discipline, in the guise of an evolution toward homogeneity and death (Prigogine and Stengers, 1979). Now, this understanding of a temporal evolution toward a state of homogenous equilibrium, equivalent to death in a living organism (that is, a return to the inanimate), is already in essence in the Freudian definition of the principle of inertia; and will find its clearest formulation with the introduction of the “death drive” in *Beyond the Pleasure Principle* (Freud, 1920).

In 1865, Rudolf Clausius (Clausius, 1865, In: Locqueneux, 2009) produced a mathematical formulation that made it possible to include both the reversible transformations of classical mechanics, as well as the irreversible physicochemical transformation that conserves energy while not being reversible (that is to say when a reversal of the functioning of the system cannot make it return to its initial state, as is the case with friction, where the motion is converted into heat) (Prigogine and Stengers, 1979). Clausius posits a state function *S*, which he calls “entropy,” so as to make a distinction between these two cases. From there Clausius concludes that the principle of conservation of energy, such as Helmholtz had recognized as a general principle, is contradicted by the second law. Thus, if the first law states that:

“A form of energy can transform into another form of energy, but the quantity of energy can never be lost; on the contrary, the total energy existing in the universe remains constant, just as the quantity of matter.” (Clausius, 1865, In: Locqueneux, 2009, p. 248)

therein proposing a concept of the universe as a whole, as absolutely irreversible, eternally performing its revolutions; the second law, that is applicable in a general way to all transformations that occur in the universe, reveals that:

“the transformations do not need to be represented in equal quantities in opposite directions, but the difference can only occur in one determinate directions […]. The outcome of this is that the state of the universe must continuously and increasingly change in one determinate direction.” (Clausius, 1865, In: Locqueneux, 2009, p. 248)

## The Application of the Principle of Entropy to the Whole Formed by the Organism and Its Environment: the Thermodynamic Origin of the Freudian Concept of “Death Drive?”

Mechanical work tends increasingly to turn into heat, there is therefore an increasing and irreversible dissipation of heat since: “heat, that constantly passes from the warmer bodies to the cooler bodies, consequently rendering the temperature equal on both sides, will gradually be distributed in an increasingly equal way; a determinate equilibrium will be established between the heat emanating from the ether, and the heat that is in the bodies” (Clausius, 1865, In: Locqueneux, 2009, p. 248). Clausius, therein, introduces the theory of a general tendency toward a state of equilibrium. A tendency where the transformations will gradually come to end in a stable state, without variations in levels, and where no further difference resulting in an effect could take place. The tendency, according to the Freudian principle of neuronal inertia, for neurons to discharge, can be assimilated to a search for a state of equilibrium, the “level = 0.”

Based on his observation of heat machines, Clausius sought to formulate as a law this progressive, diachronic, change toward a state of equilibrium, that is defined as “the state toward which the universe gradually tends.” Thus, Clausius also makes the “the vertiginous leap from motor technology to cosmology” (Prigogine and Stengers, 1979, p. 186). In this law, he gives the name “entropy” to the vastness that represents “The sum of all the transformation that must occur to bring a body or a system of bodies to its current state” (Clausius, 1865, In: Locqueneux, 2009, p. 248), and concludes from this that “in all natural phenomena, the total value of entropy can only increase without ever decreasing” (Clausius, 1865, In: Locqueneux, 2009, p. 248). He sums up this change, that constantly takes place everywhere in nature, with the following law, that has remained famous:

“the entropy of the universe tends to a maximum.” (Clausius, 1865, In: Locqueneux, 2009, p. 248)

Consequently, according to Prigogine and Stengers, Clausius introduces hereby an “arrow of time” into physics, in that entropy can only increase in the course of time or remain constant. The increase in entropy therefore translates into an irreversible temporal evolution of the system, an evolution of a spontaneous kind. Thus, for every isolated system, the future could be defined in physics as the direction in which entropy increases. The second law implies therefore that for a given isolated system, not all evolutions are of equal value: equilibrium would appear to be a veritable “attractor” for states of non-equilibrium. The irreversible increase of entropy describes a nearing of a system to a state that attracts it, that it “prefers,” and from which it no longer spontaneously distances itself; therefore, a nearing that is irreversible (Prigogine and Stengers, 1979). However, the second law does not invalidate the first law of conservation of energy; on the contrary it encompasses it in a generalized theory. Reversible changes would thus be extreme cases, in which nature has as much propensity for the initial state as for the final one (Planck, 1941).

If the universe’s entropy “tends toward its maximum” then, according to Clausius, the more the universe draws close to that limit state, the more the opportunities for new changes disappear. When that state is reached, no further change would occur, and the universe finds itself in a “persistent state of death” (Clausius, 1865, In: Locqueneux, 2009, p. 249). These considerations make it possible to reread Freud’s hypothesis of the death drive in the light of the physics model, which had from the outset been the epistemological paradigm for his initial theoretical thinking. If we apply to living organisms this tendency of the universe toward a state of equilibrium, defined by the irreversible absence of all discernible motion and all difference in tendency – in other words equivalent to a definitive death – we can envisage a closed system consisting of the unit “organism-environment.” The second law implies that within this system the different levels of energy tend toward equaling out, in such a way that the final state would be a state of equilibrium. The state toward which the system would tend would therefore be equivalent to “the reduction of the organism’s internal energy that returns it to the inorganic state” (Laplanche and Pontalis, 1967a, p. 326). Now, let us recall that in 1920, Freud describes the conservative or “regressive” character of the death drives as originating in “the coming to life of inorganic substance” (Freud, 1920, p. 44) and that it “seek(s) to restore the inanimate state” (Freud, 1920, p. 44). If the definition of the death drive as the tendency of the living organism to return to an inorganic state can, in the first instance, “seem an aberration from the point of view of the life sciences – in so far as that it seeks to infer an organism with its vital aptitudes, its adaptative functions, its energy levels, from a principle that is the negation of all constant level of difference” (Laplanche and Pontalis, 1967a, p. 328) – it appears much more coherent within a physics model. Furthermore, it should be pointed out that the first ambition of the Helmholtz School, to which Freud was heir, had as specific objective the application of the physical laws of thermodynamics to the study of living organisms.

The thermodynamics that inspired Freud described how systems reached an equilibrium characterized by a maximum of entropy. In contrast^1^, as Prigogine (1978, 1981) pointed out, when systems are far from equilibrium and driven by a large energy current, entropy may decrease and ordered patterns form. Current research focuses precisely on the self-organization of systems far from equilibrium. The neuronal dynamics, with the discharges that Freud envisaged, find echo in the theory of self-organized criticality (Bak, 1996; Vespignani and Zapperi, 1998). Major advances were achieved by adapting the evolution equations of statistical physics (Fokker–Planck equations) to out-of-equilibrium, open systems (Seifert, 2005; Tomé, 2006; Esposito et al., 2009; Jeffery et al., 2019). It is quite remarkable, in view of Freud’s affinity for Helmholtz work, that a thermodynamic potential, the free energy, appears to be the quantity that best describes the steady state of a system driven out of equilibrium because of its strong interactions with its environment (Jarzynski, 1997; Evans and Searles, 2002; Friston, 2019). Based on these new ideas, the “death drive” might be recast as a natural tendency of certain out-of-equilibrium systems to reach a steady-state characterized by a minimization of free energy. Indeed, there have been attempts to connect Freudian notions of free (unbound) energy to the variational free energy that figures in theoretical neurobiology and statistical mechanics (Carhart-Harris and Friston, 2010). If this is possible, it would mean that the death drive might now be cast in a way that is formally similar to the way Newtonian mechanics was recast in a “principle of least action.” This would constitute a major advance in Freud’s “*Project for a Scientific Psychology*.”

## Conclusion: the Death Drive: Beyond an Antithesis Between a Physical, or Biological, Paradigm

If we reconsider this idea in the light of the physiological tradition, we can point out that the Freudian death drive is not in complete opposition to the thinking of the French School. The experimental research of Claude Bernard had contributed to focusing biology away from the vitalist concept according to which (as was argued by Bichat amongst others) life should be defined as “the sum of the forces that resist death” (Bichat, 1852, p. 1). Alongside the discovery of the second law of thermodynamics, Bernardian physiology had overthrown this definition in favor of a concept of death as an integral part of the vital phenomena. Something that Bernard encapsulates in the twofold aphorism: “life is death” and “life is creation” (Bernard, 1885, p. 40), in which the two terms are indissociable and form a dialectical whole (Prochiantz, 1990). Bernard’s research on the physiology of respiration, nutrition, and organic combustion, brought to light that the destruction of tissues is the consequence of these vital functions. Thus, he rendered null all the vitalist physiology that rested on the opposition between a vital force, and a natural, physicochemical, tendency to move toward death (Prochiantz, 1990). According to Bernard, science had thus put an end to the split between two kinds of property within the living organism, the physical properties and the vital ones. Properties that were understood as being in a constant state of opposition and strife. So, no “grip” held by the vital properties within the organism, in so far as that the vital functions are regulated by a strict physicochemical determinism. Now, this critique of Bichat’s definition according to which life constitutes the sum of the forces that are in opposition to death, could, according to Prochiantz, also be interpreted as an argument against any concept of life as a singular point of resistance to the second law of thermodynamics. That is to say, as a structure that, at a given point, opposes increasing entropy (Prochiantz, 1990). Life then, would be destruction itself, compensated at each moment by the process of creation. In this respect, life could no longer be defined as that which resists destruction, or the increasing entropy of the universe if we adopt the terminology of physics. If we take into account this evolution of biology, made possible notably by the Bernardian revolution in physiology, the ideas formulated by Freud throughout his work (from the principle of inertia in 1895 up to the death drive in 1920) can, although linked to the physicalist epistemological framework, also resonate with this change in the biological understanding of living organisms. In view of these considerations they would no longer appear as an “aberration” from the view point of the life sciences, but on the contrary would revisit some of the questions raised in biology at the end of the 19th century.

## Author Contributions

JT is the main contributor of this manuscript as part of her Ph.D. thesis. J-PA, FA, and PM as supervisors, contributed to the conception and development of the research, and critically revised the manuscript for intellectual content.

## Conflict of Interest

The authors declare that the research was conducted in the absence of any commercial or financial relationships that could be construed as a potential conflict of interest.

## Footnotes

We thank the referee for suggesting to bring our historical analysis in the light of current research, part of which is the topic of the present issue.
